# Use of integrated imaging and serum biomarker profiles to identify subclinical dysfunction in pediatric cancer patients treated with anthracyclines

**DOI:** 10.1186/s40959-018-0030-5

**Published:** 2018-05-01

**Authors:** Olga H. Toro-Salazar, Ji Hyun Lee, Kia N. Zellars, Paige E. Perreault, Kathryn C. Mason, Zhu Wang, Kan N. Hor, Eileen Gillan, Caroline J. Zeiss, Daniel M. Gatti, Brooke T. Davey, Shelby Kutty, Bruce T. Liang, Francis G. Spinale

**Affiliations:** 1 Connecticut Children’s Medical Center, 282 Washington Street, Hartford, CT 06106 USA; 2 University of South Carolina School of Medicine, Columbia, SC USA; 3 Nationwide Children’s Hospital, Columbus, OH USA; 4 Yale University School of Medicine, New Haven, CT USA; 5 Jackson Laboratories, Bar Harbor, ME USA; 6 University of Nebraska, Omaha, NE USA; 7 Pat and Jim Calhoun Cardiology Center, University of Connecticut Health Center, Farmington, CT USA

**Keywords:** Anthracyclines, Cardiotoxicity, Myocardial strain, Global systolic function

## Abstract

**Background:**

Anthracycline induced cardiomyopathy is a major cause of mortality and morbidity among pediatric cancer survivors. It has been postulated that oxidative stress induction and inflammation may play a role in the pathogenesis of this process. Accordingly, the present study performed an assessment of biomarker profiles and functional imaging parameters focused upon potential early determinants of anthracycline induced cardiomyopathy.

**Methods:**

Patients (10–22 years) were prospectively enrolled between January 2013 and November 2014. Thirteen subjects completed the study and underwent serial cardiac magnetic resonance imaging and plasma biomarker profiling performed 24–48 h after the first anthracycline dose and at set dose intervals. In addition, we collected plasma samples from 62 healthy controls to examine normal plasma biomarker profiles.

**Results:**

Left ventricular ejection fraction (LVEF) decreased from 64.3 ± 6.2 at the first visit to 57.5 ± 3.3 (*p* = 0.004) 1 year after chemotherapy. A decline in longitudinal strain magnitude occurred at lower cumulative doses. A differential inflammatory/matrix signature emerged in anthracycline induced cardiomyopathy patients compared to normal including increased interleukin-8 and MMP levels. With longer periods of anthracycline dosing, MMP-7, a marker of macrophage proteolytic activation, increased by 165 ± 54% whereas interleukin-10 an anti-inflammatory marker decreased by 75 ± 13% (both *p* < 0.05). MMP7 correlated with time dependent changes in EF.

**Conclusions:**

Asymptomatic pediatric patients exposed to anthracycline therapy develop abnormal strain parameters at lower cumulative doses when compared to changes in EF. A differential biomarker signature containing both inflammatory and matrix domains occur early in anthracycline treatment. Dynamic changes in these domains occur with increased anthracycline doses and progression to anthracycline induced cardiomyopathy. These findings provide potential prognostic and mechanistic insights into the natural history of anthracycline induced cardiomyopathy.

**Trial registration number:**

NCT03211520 Date of Registration February 13, 2017, retrospectively registered.

**Electronic supplementary material:**

The online version of this article (10.1186/s40959-018-0030-5) contains supplementary material, which is available to authorized users.

## Background

Anthracycline induced cardiomyopathy (AIC), an all too common sequelae of cancer treatment, results in the development and progression of heart failure and death [1–8]. While the contributory mechanisms of AIC are multifactorial including changes in cellular oxidative stress and viability, [9–17] the structural underpinning is left ventricular (LV) remodeling and dysfunction. Past studies have provided evidence that AIC is associated with myocyte hypertrophy and structural remodeling of the myocardial extracellular matrix, whereby a loss of normal fibrillar collagen architecture is replaced by interstitial fibrosis [18–21]. A predominant pathway by which extracellular remodeling occurs is through changes in the proteolytic pathways, such as the matrix metalloproteinases (MMPs), and the local endogenous tissue inhibitors of MMPs (TIMPs) [22, 23]. Shifts in the steady-state expression of MMPs and TIMPs have been identified to occur with inflammation and cytokine release as well as with oxidative stress, [24, 25] both of which are operative in the context of AIC [18, 26–28]. However, temporal profiles of MMP/TIMPs as well as the relation to indices of inflammation have not been examined with AIC. The primary objective of this study was to examine MMP/TIMP and cytokine plasma levels in pediatric cancer patients undergoing anthracycline (AC) therapy and perform a comparative analysis with age matched reference control subjects.

Cardiac magnetic resonance (CMR), has an important role in the identification of cardiotoxicity and detection of early cardiac injury [20, 29]. A 10% reduction in ejection fraction (EF) to an EF < 55% is used to define Cancer Therapeutics-Related Cardiac Dysfunction (CTRCD) [29, 30]. Although early cardiac injury, without significant reduction in LV EF has been increasingly recognized, [20, 29, 31, 32] serial assessment of LV geometry, EF and myocardial strain and the relation to determinants of remodeling, such as MMP/TIMP profiles, has not been performed in patients undergoing anthracycline therapy. The guiding hypothesis of this study is that early changes in CMR parameters and MMP/TIMP/cytokine profiles occur in patients at increased risk for AIC.

## Methods

### Study population

**Fig. 1:**
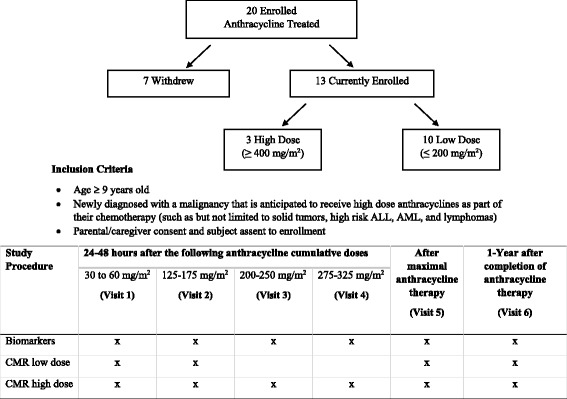
Study Cohort and Diagram. CMRI: cardiac magnetic resonance imaging; ALL: acute lymphocytic leukemia; AML: acute myeloid leukemia. Twenty patients (10–22 years) were prospectively enrolled between January 2013 and November 2014. Seventeen subjects completed visit 1 (30–60 mg/m2) and thirteen completed the remaining study visits (125–175 mg/m2, 200–250 mg/m^2^, 275–325 mg/m^2^, after max AC dose and 1 year after max AC dose)

Twenty subjects aged 10–22 years, diagnosed with cancer that required AC therapy, were prospectively enrolled between January 2013 and November 2014 at a single center. Seven subjects withdrew. Thirteen of twenty subjects enrolled completed 47 visits. The study inclusion criteria and study flow are shown in Fig. 1. CMR and biomarker studies were performed at the first visit, (V1) 30–60 mg/m^2^ cumulative anthracycline dose, and 24–48 h following set cumulative AC doses, at maximal dosing, and at 1 year after completion of AC therapy (V6)**.** Subjects were dichotomized based upon cumulative AC dose: low (< 240 mg/m^2^) or high (> 240 mg/m^2^) as described previously [33]. The medical records of all completed subjects were reviewed to identify known risk factors associated with cardiotoxicity. Conversions to isotoxic equivalents of anthracyclines were performed to calculate total cumulative dose. Sixty-two healthy human subjects (30 males and 32 females, between 9 and 35 years of age) without cardiovascular disease were enrolled to obtain normative biomarker profiles. Recruitment was performed at Connecticut Children’s Medical Center and Clinical Lab partners at the Hartford Hospital medical office building. Exclusion criteria included previous history of cardiac disease or cardiac surgery, history of chemotherapy, chronic disease or acute episode of illness (i.e. fever, upper respiratory symptoms, gastrointestinal illness, Lyme disease, mononucleosis). Demographic information for the control group is included in Table 1 and study design in Additional file 1: Figure S1.

**Table 1:** Demographics and other information of study population at first visit

	Study group (*n* = 13) Median (25–75% range) or n (%)	Subjects who withdrew (*n* = 7) Median (25–75% range) or n (%)	*p*	Control group (*n* = 62) Median (25–75% range) or n (%)	*p*
Age at diagnosis	15.7 (13.4–17.7)	15 (12–16.1)	.60	17 (13–27)	.28
Female	8 (61.5)	2 (28.6)	.35	32 (53.3)	.56
Total cumulative anthracyclines (g/m^2^)	200 (158–200)	NA		NA	
Vinca Alkaloids	9 (69.2)	NA		NA	
Previous bone marrow transplant	0 (0)	NA		NA	
Previous heart disease	3 (23.1)	NA		NA	
Height (cm)	161 (153–170.2)	NA		NA	
Weight (kg)	54 (40–84.8)	NA		NA	
BMI (kg/m^2^)	20.6 (17.9–22)	NA		NA	
Systolic pressure (mmHg)	108 (106–117)	NA		NA	
Diastolic pressure (mmHg)	64 (55–66)	NA		NA	
Number of transfusions (*n* = 11)	10 (0–49)	NA		NA	
Cancer Diagnosis			.03	NA	
ALL	4 (30.8)	0 (0)		NA	
Hodgkin’s Lymphoma	6 (46.2)	1 (14.3)		NA	
AML	1 (7.7)	2 (28.6)		NA	
Osteosarcoma	2 (15.4)	1 (14.3)		NA	
Other	0 (0)	3 (42.9)		NA	

*n* number of subjects, *SD* standard deviation, *BMI* body mass index, *ALL* acute lymphoblastic leukemia, *AML* acute myeloblastic leukemia

All p-values are compared to the study group (*n* = 13) only

### CMR techniques

CMR techniques have been previously reported [20]. Subjects were imaged on a 1.5 Tesla (GE CV software version 16.0/M4, Milwaukee, WI) with the following protocol: (1) Standard multi-slice, multi-phase cine imaging using a steady state free precession acquisition technique (Fast Imaging Employing Steady-state Acquisition, FIESTA) in the 2 chamber, 4 chamber and contiguous short axis planes. (2) Tagged cine CMR acquired in the 4-chamber and three short-axis planes (basal, mid-cavity, and apical) with an ECG-triggered segmented k-space fast gradient echo sequence with spatial modulation of magnetization in orthogonal planes. (3) Gadopentetate dimeglumine DTPA (Magnevist; Schering, Berlin, Germany) was administered intravenously at a dose of 0.1 mmol per kilogram of body weight. (4) Late Gadolinium enhancement (LGE) was acquired 10–15 min after intravenous contrast for assessment of signal intensity changes in the 4-chamber and contiguous short axis planes. TI (time to inversion) was calculated to null the myocardium.

### Image analysis

LV volumes, mass, and global function were measured via standard planimetry techniques with semi-automated computer software (QMASSversion 6.1.5, Medis Medical Imaging Systems, Leiden, the Netherlands) by an experienced reader (PI). Tagged images were analyzed with the HARmonic Phase (HARP, Diagnosoft, Palo Alto, California) technique [34] for calculation of peak average circumferential (εcc) and longitudinal strain (ειι). The CMR studies were de-identified and volumetric and HARP analysis of strain performed blindly by two experienced investigators (OTS, KNH).

### Methods for biomarkers

Blood was collected from subjects, immediately centrifuged and the plasma layer removed. The separated plasma was divided into three equal aliquots and frozen at − 70 °C. The following markers were measured: Matrix/Fibrosis pathway: Plasma levels of MMPs (all soluble MMP types) and TIMPs (all 4 TIMPs); Inflammatory domain: cytokines (TNFα, interleukins, interferon gamma (IFNG), TGF β2, TGF βII)), cytokine receptors (sTNF RI, sTNF RII, sSt2, sgp130, siL1RII/sCD121b, siL-2Rα/CD25, siL-4R, siL-6R, Endoglin); Signaling pathway: growth factors (GDP-15, GCSF, VEGF, sVEGFR2 TGFβ1, IGF-1; The specific antisera and sensitivity for this approach is provided in Additional file 2: Table S1. All measurements were performed in duplicate using an internally validated and calibrated multiplex suspension array (two-laser flow cytometric detection system; Bio-Plex 200, BioRad Laboratories). The sensitivity for all of the assays was in the pg/mL range, with intra-assay coefficient of variations less than 15%.

### Statistical analysis

Data are expressed as numbers with percentages and median values with ranges (25%–75%), as appropriate. The Wilcoxon rank sum test was conducted to examine differences between study group and control group. Student’s t-tests were used to examine differences between groups: high dose vs. low dose, and the first visit values compared to maximal dose and 1 year after chemotherapy, and to compare biomarker concentrations between subjects and controls. Spearman correlation analysis was conducted to assess the relationship between continuous variables. Non-parametric test for trend on MRI parameters across all visits was conducted and *p*-values are presented in Table 2. All tests were two-tailed, and *p* < 0.05 was considered to indicate a statistically significant difference. Stata 14.2 (StataCorp, Texas USA) software was used for statistical analysis. To evaluate associations of biomarkers and time dependent changes in EF from V1 to V6, we utilized multilevel modeling for repeated measures (R program v 3.4.0, R Foundation for Statistical Computing, Vienna, Austria). In the multilevel modeling for each biomarker, an overall linear change function is fitted to the whole sample and the slope and intercept are allowed to vary across individuals, i.e. defined as random coefficients. We report fixed regression coefficients that represent the associations between biomarkers and time dependent changes in EF from V1 to V6 averaged across subjects. Given the small sample size, we did not adjust p-values for multiple comparisons.

**Table 2:** LV Parameter trends (medians) by Cardiac Magnetic Resonance Imaging

Cumulative AC Dose (mg/m^2^)	High dose group (*n* = 3)							Low dose group (*n* = 10)				
	30–60	125–175	200–250	275–325	After Max	1 Year After	*p*	30–60	125–175	After Max	1 Year After	p
LV EF (%)	66.8	61.2	58.7	57.6	55.8	55.3	**.005**	62.6	60.8	60.4	57.9	.11
LV EF z score	0.57	−0.62	−1.1	−1.4	−1.7	−1.9	**.005**	−0.3	−0.6	−0.8	−1.3	.11
LV EVDI (ml/m^2^)	96.6	85.3	100.6	97.1	74.8	94.1	.22	75.6	78.3	73.9	78.3	.56
LV ESVI (ml/m^2^)	23.5	33.2	40.3	41.1	31.8	41.5	.30	29.3	30.7	29.9	33.0	.58
LV SV (ml)	132.3	88.5	87	85.6	77.5	99.2	.35	73.4	67.5	73.9	67.6	.93
LV Mass (g/m^2^)	133	107.1	135	128	122	135.8	.62	88.9	79.0	83	87.0	.87
LV Mass z score	0.4	0.7	0.2	0.5	0.3	0.6	.42	0.6	0	−0.2	−0.2	**.03**
LV Mass Volume (g/ml)	0.7	0.8	0.8	0.8	0.9	0.8	.26	0.8	0.8	0.7	0.7	.19
LV Mass Volume z score	−1.0	0.5	−0.3	−0.5	0.9	−0.4	.54	0.5	0.7	0.3	0.2	.28
ESFS (g/cm^2^)	99	89.5	115	115	114	122	**.02**	109	107.5	99	104	.97
ESFS z score	2.2	1.4	3.67	3.63	3.45	4.18	**.02**	3.3	3.1	2.3	2.8	.83
εcc (%)	−22.5	−20.3	−19.1	−18.1	−16.3	−18.9	**.02**	−21.1	−19.0	−19.3	− 19.9	.45
ειι (%)	− 19.1	−19.3	− 16.2	−15.8	− 14.7	−17.4	**.02**	−18.0	− 16.0	−15.5	− 16.4	.56

*LV* left ventricular, *CMR* cardiac magnetic resonance imaging, *n* number of subjects, *AC* anthracycline, *EF* ejection fraction, *EDVI* end diastolic volume index, *ESVI* end systolic volume index, *SV* stroke volume, *ESFS* end systolic fiber stress; *εcc* peak global circumferential strain magnitude; ειι: Peak global longitudinal strain magnitude. All patients in the low dose group had diagnosis of leukemia or lymphoma with Anthracycline cumulative doses of 175.2 ± 21.4 compared to 447.3 ± 4.6 (*p* < .0001) in the high dose group (solid tumor diagnosis)

Bold numbers indicate statistically significant cardiac magnetic resonance imaging parameters

## Results

The clinical and demographic characteristics for thirteen of twenty subjects who completed all visits is shown in Table 1. Changes in imaging parameters 24–48 h after AC dose at all visits in high and low dose subjects are shown in Table 2. All subjects in the low dose group had diagnosis of leukemia or lymphoma with AC doses of 175.2 ± 21.4 compared to 447.3 ± 4.6 (*p* < .0001) in the high dose group (solid tumor diagnosis).

### Imaging parameters

Using CMR we compared the effect of AC therapy on LVEF 24–48 h following 30–60 mg/m^2^ of anthracyclines (first visit), after maximal therapy and 1 year after maximal therapy. The LVEF decreased from 64.7 ± 6.1 (Z score: 0.1 ± 1.3) after the initial dose (V1) to 59.0 ± 7.0 (Z score: − 1.1 ± 1.5), *p* = 0.04 (Z score *p* = 0.05) at the end of maximal therapy. The mean decrease in EF was statistically significant when we compared LVEF between the first visit and 1 year after maximal therapy.

(6.76 ± 6.5, Z score: 1.4 ± 1.4, *p* = 0.004; *p* = 0.005, respectively). Seven subjects demonstrated greater than 10% absolute unit reduction in EF from initial visit to maximal therapy. Three (23.1%) of these subjects met criteria for CTRCD (cancer therapeutic related cardiac dysfunction) with > 10% reduction to an EF < 55% at maximal therapy. Two of three subjects had a persistent reduction in EF 1 year after chemotherapy.

To better characterize early cardiotoxic effects of AC in the heart we then determined regional myocardial function by measuring average εcc and ειι. Medians of LV parameters including strain values are included in Table 2 for all time visits.

### Regional myocardial function (strain)

**Fig. 2:**
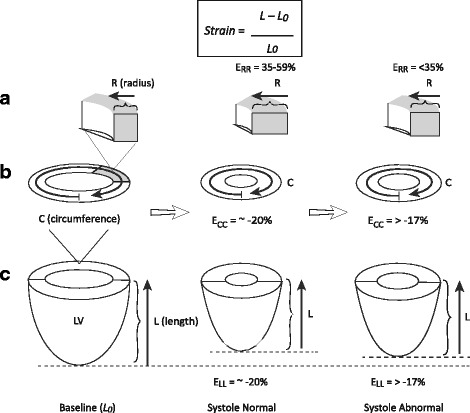
Schematic diagram demonstrating the three dimensional circumferential-radial-longitudinal coordinate system used for strain calculation. E_RR;_ Peak mid radial strain magnitude; E_CC_: Peak global circumferential strain magnitude; ELL: Peak global longitudinal strain magnitude. Each element of strain is, simply, a measurement of the fractional or the percent change of length in a specific direction where Lo is the original fiber length before tag deformation and L is the current length

Complex fiber architecture in the heart results in complex patterns of deformation and changes in shape that are produced upon muscle contraction or relaxation. Each element of strain is simply a measurement of the fractional or the percent change of length in a specific direction where L_o_ is the original fiber length before tag deformation and L is the current length (Strain = L- L_o_/L_o_). In a segment, radial strain (ε_*RR*_) describes myocardial thickening, which is in the radial direction towards the center of the ventricle. (ε_*RR*_) has high variability due to the impact of circumferential and longitudinal strain and thus has not been routinely used (Fig. 2a). Circumferential strain (εcc) of the same segment describes circumferential shortening, which is a direction tangential to the epicardial surface (Fig. 2b). The third strain component in a segment is longitudinal strain (ειι), which represents base-to-apical shortening along the ventricular long axis (Fig. 2c) [35]. The depreciation of the strain magnitude, or percent change, indicates the weakening of the myocardial in a segment. The average of segmental strain measurements over the ventricle is a measure of global strain. Global longitudinal strain (ειι), is currently considered the optimal parameter of deformation for the early detection of subclinical dysfunction in CTRCD [30]. For longitudinal and circumferential strain, a less negative value constitutes a reduction in strain magnitude (Fig. 2b and c). An absolute reduction in strain to > − 17% and /or a relative percentage decrease of > 15% compared with baseline is likely to be of clinical significance. LV parameters including strain are included in Table 3 for all time visits.

**Table 3:** Multilevel linear regression (biomarkers to changes in LV Ejection Fraction from V1 to V6)

	Coef.	SE	DF	t	p
εcc (%)	−1.5540	0.3924	35	−3.9605	<.001
ESV (ml)	−0.2496	0.0446	43	−5.5980	<.001
ESFS (g/cm^2^)	−0.1584	0.0451	43	−3.5114	.0011
MMP7 (pg/ml)	−0.0014	0.0006	43	−2.4592	.018
sIL 4R (pg/ml)	0.0034	0.0014	44	2.4405	.0188
sRage (pg/ml)	−0.0502	0.0216	44	−2.3234	.0248
sTNFRI (pg/ml)	−0.0032	0.0015	44	−2.1340	.0385
sTNFRII (pg/ml)	−0.0008	0.0003	44	−2.4872	.0167
sVEGFR3 (pg/ml)	−0.0058	0.0022	44	−2.6162	.0121

V1: first visit; V6: 1 year after maximal anthracycline therapy; εcc: Peak global longitudinal strain magnitude; ESV: End systolic volume; ESFS: End systolic fiber stress; MMP: Metalloproteinase; sILR: soluble interleukin receptor; sRage: receptor for advanced glycation end products; sTNFR: soluble receptor for TNF; sVEGFR: receptors for vascular endothelial growth factor

### High dose group (mean AC cumulative dose 447.3 ± 4.6 mg/m^2^) *n* = 3

There was a decrease in εcc magnitude from − 22.2 ± 0.7 after 30–60 mg/m^2^ cumulative AC dose to − 17.1 ± 1.6 (*p* = 0.02) at maximal dose and a clinically significant decrease in ειι magnitude from-19.5 ± 0.9 to − 15.8 ± 2.8 (*p* = .20). A clinically significant decline in ειι magnitude (− 17.2 ± 1.9, *p* = 0.25) occurred at lower cumulative doses (200–250 mg/m^2^) followed by a decline in εcc magnitude (− 17.8 ± 2.6, *p* = 0.07) at 275–325 mg/m^2^ and EF < 55% at doses > 375 mg/m^2^ (Table 2).

### Low dose group (mean AC cumulative dose 175.2 ± 21.4 mg/m^2^) *n* = 10

There was a decrease in ειι magnitude from − 17.1 ± 2.6 to − 15.1 ± 4.9 at maximal therapy (*p* = 0.44) and no significant change in εcc magnitude from − 20.7 ± 1.0 at V1 to − 20.3 ± 3.1 at maximal therapy (*p* = 0.77).

### Biomarker analysis

#### The extracellular matrix (ECM) biomarker profiles

**Fig. 3:**
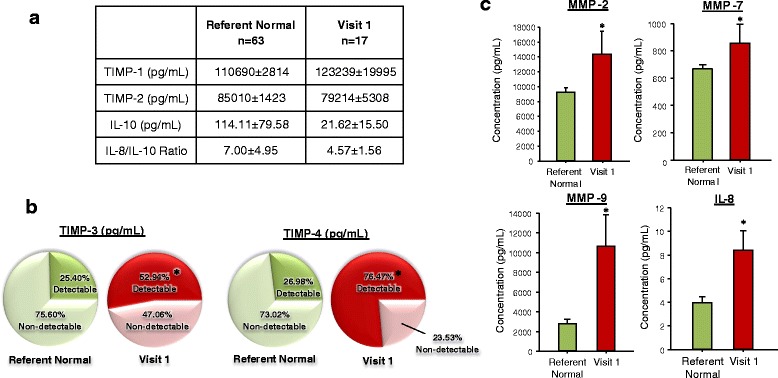
Plasma profiling for specific determinants of inflammation and signaling. **a** Plasma profiling for specific determinants of inflammation and ECM remodeling were examined in referent normal subjects and those undergoing initial AC treatment, designated here as Visit 1 in tabular format. The plasma TIMP-1 and 2 levels at this AC treatment time point were not different from referent controls, and while IL-10 and IL-8/IL-10 ratios fell in the AC treatment group at Visit 1, these did not reach statistical significance. **b** Due to the high incidence of plasma TIMP-3 and TIMP-4 levels were fell below analytical detection limits, a categorical analysis was performed using a Chi Square analysis. A significant shift in the proportion of detectable TIMP-3 and TIMP-4 in plasma samples taken from the initial AC treatment time point occurred (*p < 0.05). **c** In contrast to the analyte shown in Table A, significant shifts in plasma MMP-2, − 7 and − 9 occurred at AC treatment Visit 1 when compared to referent control values (*p < 0.05)

The matrix metalloproteinases (MMPs) play a key role during cardiac remodeling [36]. Cardiac matrix alterations induced by anthracyclines [19] and strong transcriptional activation for several specific MMPs in anthracycline-induced cardiac remodeling has been demonstrated in several animal models [21, 37, 38]. We demonstrate an increase in MMP-2, MMP-7 and MMP-9, and detectable levels of TIMP-3 and TIMP-4 in the anthracycline group (*p* < 0.05) (Fig. 3).

### Inflammation related biomarker profiles

**Fig. 4:**
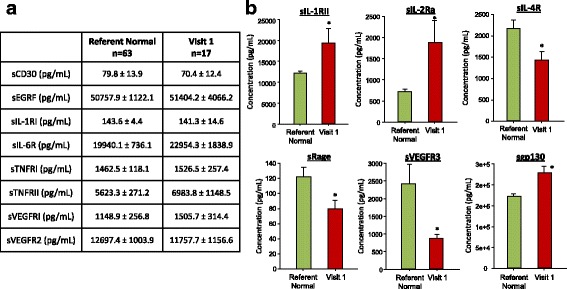
Plasma profiling for specific determinants of inflammation and signaling. A number of inflammatory signaling pathways were profiled in plasma samples taken from age matched referent normal subjects and at the initial visit following AC treatment- identified as Visit 1. While robust signals for the soluble IL, TNF and VEGF receptors were detected in both referent normal and Visit 1 samples many soluble receptor analytes were similar between groups and summarized in Table A. On the other hand, specific soluble receptors/pathways were significantly different from referent control values and are shown in Figure 2-2B. These included the soluble receptors for IL-1, -2, and -4. A relative reduction in sRage and sVEGFR3 occurred in Visit 1 samples with an increase in Sgp130. (*p < 0.05 vs Visit 1 values). Normal: referent normal; MMP-2: Matrix metallopeptidase 2; MMP-7: Matrix metallopeptidase 7; MMP-9: Matrix metallopeptidase 9; IL-8: Interleukin 8; sVEGFR3: Fms-related tyrosine kinase 4; sgp130: Interleukin 6 signal transducer; sIL-1RII: Interleukin 1 receptor, type II; sIL-2Ra: Interleukin 2 receptor, alpha; sIL-4R: Interleukin 4 receptor

Cytokines are a family of bioactive signaling molecules that regulate the inflammatory response and have been recently identified as mediators in the development and progression of heart failure [39, 40]. These inflammatory mediators are now known to be expressed by all nucleated cell types residing in the myocardium, including the cardiac myocyte [41]. We used a multiplex array panel to quantify signatures from the inflammatory domain (cytokines, cytokine receptors) and signaling pathways. Statistically significant elevation in IL-8, IL-1 and IL-2 receptors (sIL-1RII, sIL-2Ra) and soluble glycoprotein (sgp) 130, were observed in subjects 24–48 h after 30–60 mg/m^2^ of AC therapy when compared to controls (Figs. 3 and 4). Similarly, there was a statistically significant early down-regulation of soluble IL-4 receptor (siL4R), the vascular endothelial growth factor (VEGF), and receptor for advanced glycation end products (sRage) (Fig. 4).

**Fig. 5:**
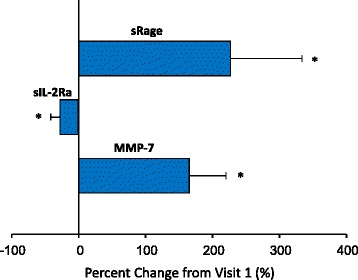
Plasma profiling changes from Visit 1 to last available visit in AC treated patients. The relative change in ECM and inflammatory pathways were computed as a function of Visit 1 and the final visit in the AC treated patients. Those with a significant shift from Visit 1 are shown here and identify that continued shifts in determinants of inflammatory signaling and ECM remodeling occurred at later AC treatment time points. (*p < 0.05 vs Visit 1 values)

With longer periods of AC dosing, MMP-7, a marker of macrophage proteolytic activation, increased by 165 + 54% whereas interleukin-10 (IL-10) an anti-inflammatory marker fell by 75 + 13% (both *p* < 0.05) (Fig. 5).

### Relation of LV dysfunction and biomarker profiles

Having demonstrated an early (30–60 mg/m^2^) differential inflammatory signature and ECM biomarker profile in AIC patients, we then correlated them with regional and global parameters of myocardial function. Elevations in IL-8 at the first visit correlated with lower εcc after maximal therapy (*r* = − 0.61, *p* = .04) and 1 year after chemotherapy (*r* = − 0.73, *p* = 0.008). Low IL-10 at first visit was associated with a low ειι 1 year after chemotherapy (*r* = − 0.82, *p* = 0.09) and > 10% decrease in EF from V1 to V6.

Early elevation in MMP7 levels (30–60 mg/m2) correlated with a > 10% decrease in EF from V1 to V6. (Table 3 and Additional file 2: Table S2). Early down regulation in several soluble receptors, including, siL4R, sRage, sTNFR1, sTNFRII and sVEGFR3 correlated with time dependent changes in EF from V1 to V6.

## Discussion

This pilot study is a breakthrough in the use of serial combined functional imaging-serum biomarker profiling in a cohort of subjects undergoing AC chemotherapy. Decrease in CMR–derived εcc and ειι, and increase and serologic biomarkers of matrix/fibrosis pathways, inflammatory domain cytokines, cytokine receptors and signaling pathways occur early in the course of anthracycline therapy. With higher AC cumulative doses, MMP-7, a marker of macrophage proteolytic activation increases, whereas there is a decrease in IL-10 an anti-inflammatory marker. Moreover, we demonstrate a subgroup of these biomarkers are associated with changes in EF from V1 (30–60 mg/m^2^ AC dose) to V6 (1 year after chemotherapy). This biomarker signature may provide insights into the pathogenic mechanisms underlying cardiac remodeling and the transition to chronic delayed cardiotoxicity.

Early reduction in myocardial strain or strain rate magnitude using echocardiography has been shown to predict subsequent cardiotoxicity [30, 42, 43]. A relative percentage reduction of global longitudinal strain (GLS) of > 15% from baseline appears to be meaningful, and is likely to be abnormal [44]. Similarly, CMR-based spatial modulation of magnetization has shown a decline of mid-wall circumferential strain at 1 month of AC therapy, which remained reduced at 6 months [31]. This is the first study to demonstrate that changes in longitudinal and circumferential strain magnitude by CMR occur before changes in LVEF with increasing doses of anthracyclines. Furthermore, early measurement of regional deformation parameters (30–60 mg/m^2^) correlate with a decrease in EF > 10% from V1 to V6.

Infiltration of the myocardium in acute and chronic leukemia has been reported previously [45]. Lower strain values at the first visit (30–60 mg/m^2^) were noted in patients with leukemia and lymphoma, when compared to those with solid tumors. AC doses were significantly lower in this group (175.2 ± 21.4 compared to 447.3 ± 4.6 < .0001). Early decrease in myocardial deformation parameters in these patients is suggestive of baseline involvement of the myocardium related to their cancer diagnosis. Of significance, leukemia and lymphoma patients with the lowest strain values at first visit had the lowest EF after completion of AC therapy.

### Inflammation and relevance for biomarker profiling

Inflammatory mediators play an important role in LV remodeling. Major steps in this process include myocyte hypertrophy, [46] alterations in fetal gene expression, [47] progressive myocyte loss through apoptosis [48] and alterations in the extracellular matrix leading to microscopic fibrosis [17]. These inflammatory mediators are known to be expressed by all nucleated cell types residing in the myocardium [40]. Several studies have shown raised levels of inflammatory cytokines, such as tumor necrosis factor (TNF), interleukins from the IL-1 and IL-6 families in plasma, and circulating leukocytes in heart failure patients [49]. Pro-inflammatory cytokines including TNF, IL-1β, and IL-6 are upregulated with decreased heart function, and are activated earlier in heart failure than the classic neurohormones [50]. We demonstrated an early increase in IL-8 and a decrease in the IL-8/IL-10 ratio in children with AIC, indicating pro-inflammatory activity. Similar findings have been reported in patients with heart failure [51] and in pediatric patients undergoing open heart surgery [52]. While soluble ligands circulate at low levels, their corresponding receptors are frequently detected at high levels in plasma, suggesting these receptors have potential as reliable biomarkers. We observed increased levels of soluble IL-1 and IL-2 receptors early in the course of AC therapy. IL-2, a human recombinant product, effective in the treatment of a variety of malignancies including neuroblastoma, has been associated with important treatment-related clinical toxic effects [53]. IL-2 toxicity can manifest in multiple organ systems, most significantly the heart, lungs, kidneys, and central nervous system [54]. Larger studies may help us understand how AC modulates cytokine production in relation to cardiotoxicity.

Additionally, we observed increased levels of soluble glycoprotein (sgp) 130, a common signal-transducing receptor of the IL-6 family known to be associated with mortality in congestive heart failure patients [49]. The early down-regulation of VEGF and receptor for advanced glycation end products (sRage) found in our study suggests involvement of cell growth/viability pathways responsible for vascular remodeling and myocardial angiogenesis [55, 56]. Taken together, this data strongly indicates a role for inflammation in AIC and reveals potentially robust set of biomarkers for monitoring pediatric AIC.

### Extracellular matrix biomarker profiles

Matrix metalloproteinases (MMPs) are a family of proteolytic enzymes responsible for the breakdown of the extracellular matrix [57]. Naturally occurring tissue inhibitors of MMPs (TIMPs) can partially or completely neutralize MMP function [58]. MMPs are upregulated in heart failure, and an increased myocardial MMP/TIMP ratio results in altered extracellular matrix architecture and adverse remodeling, leading to ventricular dilatation and dysfunction [23, 59–63]. Remodeling with loss of extracellular matrix has been demonstrated to continue for weeks after a single treatment of anthracyclines [19]. We demonstrated an early increase in MMP-2, MMP-7 and MMP-9 and detectable levels for TIMP-3 and TIMP-4 in the anthracycline group. With longer periods of AC dosing, MMP-7, a marker of macrophage proteolytic activation, increased by 165 + 54%. Macrophages control processes of inflammation, cardiac remodeling and healing following acute coronary event, and may play a role in the pathogenesis of AIC [64].

Enhancement of MMP-2 and MMP-9, both gelatinases involved in the degradation of collagen type IV, has been shown in early AIC in h9c2 cells [38]. Strong activation of MMP-1, MMP-2, MMP-9 and a weaker induction of TIMP-1 have also been demonstrated in a porcine model [37]. Disproportionate MMP inhibitor activity may result in excessive scar formation that may convert systolic heart failure into diastolic heart failure through fibrosis and collagen deposition [22, 65].

#### Mechanistic insights and future directions

Structural changes at the time of LV assist device implantation in heart failure patients who had been exposed to AC, have demonstrated varying degrees of adverse cardiac remodeling with moderate-to-severe myocyte hypertrophy, moderate myocytolysis, and perivascular and interstitial fibrosis with areas of replacement fibrosis [66]. Prolonged hypertrophy and fibrosis in response to pathological signals is associated with an increase in morbidity and mortality as a result of impaired left ventricular relaxation and systolic dysfunction with development of heart failure. Insights into the regulatory pathways responsible for AIC and the development of a predictive biomarker signature for early detection of myocardial dysfunction may allow early identification of patients who are most at risk for severe toxicity, as well as the evaluation of new therapies to prevent heart failure. Future innovative translational and clinical approaches, including functional imaging, biomarker and genetic profiling, will provide potential prognostic and mechanistic insights into the progression of AIC.

## Conclusions

Asymptomatic pediatric patients exposed to AC chemotherapy develop myocardial strain magnitude abnormalities at low cumulative doses, with a decline in ειι preceding changes in EF. A differential biomarker signature comprised of both inflammatory and matrix domains occurs early in the treatment course. This early biomarker signature correlates with decrease in EF with increasing dose of anthracyclines. Future studies with larger number of patients will help validate our findings and may provide prognostic and mechanistic insights into the progression of AIC.

### Limitations

Because of the small sample size, most of our results are descriptive, with statistically meaningful conclusions only for differences that are extremely large. Given the small sample size we were not able to not adjust *p*-values for multiple comparisons. Obtaining baseline CMR and biomarkers prior to dosing may help differentiate effect of cancer and response to chemotherapy from cardiotoxic effects and should be addressed in future studies. Although echocardiograms were performed at all visits, we chose strain calculations with HARP imaging by CMR since this technique is considered the gold standard for the measurement of regional myocardial deformation [35, 67–69]. We previously reported 2D and 3D speckle-tracking echocardiography (STE) based measurements of GLS magnitude were highly specific in identifying subjects with abnormal longitudinal strain magnitude by CMR with specificity and positive predictive value of 92% while 2D and 3D STE-derived GCS values were not predictive of decreased peak circumferential strain magnitude (εcc) by CMR [20]. Maintaining patient compliance with the study was difficult due to the complicated treatment course in some patients, which resulted in 7 withdrawals. Newer techniques such as Strain-encoding (SENC) and DENSE (Displacement Encoding with Stimulated Echoes) MRI coupled with compressed sensing techniques will facilitate the performance of short, breath-through MRI studies for accurate estimation of parameters of regional and global myocardial function.

## Additional files

Additional file 1: Normal Control Cohort. (DOCX 22 kb)

Additional file 2: **Table S1.** Plasma analytes assessed in referent normal subjects and in patients undergoing anthracycline therapy. **Table S2.** Linear regression of biomarkers at V1 to changes in Ejection Fraction > 10% from V1 to V6). (PDF 186 kb)

## Abbreviations

AC: Anthracycline
AIC: Anthracycline induced cardiomyopathy
CMR: Cardiac magnetic resonance imaging
EF: Ejection fraction
ESFS: End systolic fiber stress
LV: Left ventricular
MMP: Metalloproteinase
ROS: Reactive oxygen species
TIMP: Inhibitors of metalloproteinase
εcc: Peak global circumferential strain magnitude
ειι: Peak global longitudinal strain magnitude
