# Supplementary material for: Use of integrated imaging and serum biomarker profiles to identify subclinical dysfunction in pediatric cancer patients treated with anthracyclines
Source: Cardiooncology. 2018 May 1;4:4. doi: 10.1186/s40959-018-0030-5 (PMC5995570; doi:10.1186/s40959-018-0030-5)
Supplement: Supplementary file 1 — Normal Control Cohort. (DOCX 22 kb) [file 40959_2018_30_MOESM1_ESM.docx]

Additional file 1

Age 9-17

18 males and 14 females

62 enrolled

Age 18-35

12 males and 18 females

**Inclusion Criteria**

- Healthy individuals between the ages of 9 and 35 years with no prior history of heart disease or heart failure

**Exclusion Criteria**

- Subjects who are unable to give informed consent.
- Subjects who have previous history of cardiac disease or cardiac surgery
- Subjects who have a fever and/or upper respiratory symptoms
- Subjects who are or have received chemotherapy
- Subjects with any history of chronic inflammatory disease
- Subjects with an acute episode of illness (i.e., Lyme disease, mononucleosis, gastrointestinal illness, etc.)

**Figure S1. Normal Control Cohort**
