# Supplementary material for: Use of integrated imaging and serum biomarker profiles to identify subclinical dysfunction in pediatric cancer patients treated with anthracyclines
Source: Cardiooncology. 2018 May 1;4:4. doi: 10.1186/s40959-018-0030-5 (PMC5995570; doi:10.1186/s40959-018-0030-5)
Supplement: Supplementary file 2 — Table S1. Plasma analytes assessed in referent normal subjects and in patients undergoing anthracycline therapy. Table S2. Linear regression of biomarkers at V1 to changes in Ejection Fraction > 10% from V1 to V6). (PDF 186 kb) [file 40959_2018_30_MOESM2_ESM.pdf]

Supplemental Table 1. Plasma analytes assessed in referent normal subjects and in patients undergoing anthracycline therapy

Human Soluble Cytokine Receptor Multiplex Panel					
**Abbreviation**	**Full name**	**Mean sensitivity (minimum detectable concentration, pg/ml)**	**Serum Dilution Factor**	**Catalog #**	**Manufacturer**
sCD30	Tumor necrosis factor receptor superfamily, member 8	7.0	1:5	HCSRMAG-32K	Millipore
sEGFR	Epidermal growth factor receptor	42.0	1:5	HCSRMAG-32K	Millipore
sgp130	Interleukin 6 signal transducer	6.0	1:5	HCSRMAG-32K	Millipore
sIL-1RI	Interleukin 1 receptor, type I	21.0	1:5	HCSRMAG-32K	Millipore
sIL-1RII	Interleukin 1 receptor, type II	115.0	1:5	HCSRMAG-32K	Millipore
sIL-2Rα	Interleukin 2 receptor, alpha	11.0	1:5	HCSRMAG-32K	Millipore
sIL-4R	Interleukin 4 receptor	14.0	1:5	HCSRMAG-32K	Millipore
sIL-6R	Interleukin 6 receptor	9.0	1:5	HCSRMAG-32K	Millipore
sRAGE	Advanced glycosylation end product-specific receptor	3.0	1:5	HCSRMAG-32K	Millipore
sTNFRI	Tumor necrosis factor receptor superfamily, member 1A	12.0	1:5	HCSRMAG-32K	Millipore
sTNFRII	Tumor necrosis factor receptor superfamily, member 1B	8.0	1:5	HCSRMAG-32K	Millipore
sVEGFR1	Fms-related tyrosine kinase 1	111.0	1:5	HCSRMAG-32K	Millipore
sVEGFR2	Kinase insert domain protein receptor	71.0	1:5	HCSRMAG-32K	Millipore
sVEGFR3	Fms-related tyrosine kinase 4	47.0	1:5	HCSRMAG-32K	Millipore

Human Cytokine Multiplex Panel					
**Abbreviation**	**Full name**	**Mean sensitivity (minimum detectable concentration, pg/ml)**	**Serum Dilution Factor**	**Catalog #**	**Manufacturer**
GM-CSF	Granulocyte macrophage colony-stimulating factor	0.2	1:4	M50000007A	Biorad
IFN-γ	Interferon gamma	6.4	1:4	M50000007A	Biorad
IL-2	Interleukin 2	1.6	1:4	M50000007A	Biorad
IL-4	Interleukin 4	0.7	1:4	M50000007A	Biorad
IL-6	Interleukin 6	2.6	1:4	M50000007A	Biorad
IL-8	Interleukin 6	1.0	1:4	M50000007A	Biorad
IL-10	Interleukin 10	0.3	1:4	M50000007A	Biorad
TNF-α	Tumor Necrosis Factor alpha	6.0	1:4	M50000007A	Biorad

**Human Matrix Remodeling Multiplex Panel**					
**Abbreviation**	**Full name**	**Mean sensitivity (minimum detectable concentration, pg/ml)**	**Serum Dilution Factor**	**Catalog #**	**Manufacturer**
TIMP1	TIMP metallopeptidase inhibitor 1	1.6	1:50	171-AM002M	Biorad
TIMP2	TIMP metallopeptidase inhibitor 2	2.4	1:50	171-AM002M	Biorad
TIMP3	TIMP metallopeptidase inhibitor 3	96.3	1:50	171-AM002M	Biorad
TIMP4	TIMP metallopeptidase inhibitor 4	1.7	1:50	171-AM002M	Biorad
MMP1	Matrix metallopeptidase 1	35.0	1:10	171-AM001M	Biorad
MMP2	Matrix metallopeptidase 2	450.0	1:10	171-AM001M	Biorad
MMP3	Matrix metallopeptidase 3	116.0	1:10	171-AM001M	Biorad
MMP7	Matrix metallopeptidase 7	5.4	1:10	171-AM001M	Biorad
MMP8	Matrix metallopeptidase 8	1.5	1:10	171-AM001M	Biorad
MMP9	Matrix metallopeptidase 9	24.0	1:10	171-AM001M	Biorad
MMP10	Matrix metallopeptidase 10	1.6	1:10	171-AM001M	Biorad
MMP12	Matrix metallopeptidase 12	1.0	1:10	171-AM001M	Biorad
MMP13	Matrix metallopeptidase 13	4.9	1:10	171-AM001M	Biorad

Human Cardiac Troponin I ELISA					
**Abbreviation**	**Full name**	**Mean sensitivity (minimum detectable concentration, pg/ml)**	**Serum Dilution Factor**	**Catalog #**	**Manufacturer**
cTnI	Cardiac Troponin I	100	1:2	ELH-CTNI	RayBiotech

Supplemental Table 2. Linear regression of biomarkers at V1 to changes in Ejection Fraction > 10% from V1 to V6)

	Coef.	SE	DF	t	p
Ɛcc (%)	-1.5540	0.3924	35	-3.9605	**<.001**
ESV (ml)	-0.2496	0.0446	43	-5.5980	**<.001**
EDV (ml)	-0.0187	0.0268	43	-0.6980	.4889
Mass (g)	-0.0459	0.0425	43	-1.0786	.2868
Mass Volume (g/ml)	-5.2095	8.7259	43	-0.5970	.5536
ESFS (g/cm^2)	-0.1584	0.0451	43	-3.5114	**.0011**
BSA (m^2)	-4.0437	3.5390	43	-1.1426	.2595
CTnI (pg/ml)	0.0003	0.0002	44	1.8203	.0755
TIMP1 (pg/ml)	0.0000	0.0000	43	-0.6539	.5167
TIMP2 (pg/ml)	-0.0001	0.0000	43	-1.9313	.0601
TIMP3 (pg/ml)	0.0000	0.0002	43	-0.1691	.8665
TIMP4 (pg/ml)	0.0008	0.0004	43	2.0004	.0518
MMP1 (pg/ml)	0.0017	0.0016	43	1.0343	.3068
MMP2 (pg/ml)	0.0000	0.0001	43	-0.4870	.6287
MMP3 (pg/ml)	0.0000	0.0000	43	0.4706	.6403
MMP7 (pg/ml)	-0.0014	0.0006	43	-2.4592	**.018**
MMP8 (pg/ml)	0.0002	0.0003	43	0.5972	.5535
MMP9 (pg/ml)	-0.0001	0.0001	30	-0.9421	.3537
MMP13 (pg/ml)	-0.0205	0.0118	43	-1.7378	.0894
GM.CSF (pg/ml)	-0.0030	0.0367	43	-0.0805	.9362
IFNɣ (pg/ml)	0.3151	3.1086	43	0.1014	.9197
IL4 (pg/ml)	-11.0271	438.9031	43	-0.0251	.9801
IL8 (pg/ml)	0.0003	0.1299	43	0.0026	.9979
sCD30 (pg/ml)	-0.0145	0.0240	44	-0.6058	.5478
sEGRF (pg/ml)	-0.0001	0.0001	44	-1.8501	.071
sIL 1RI (pg/ml)	-0.0182	0.0203	44	-0.8946	.3758
sIL 1RII (pg/ml)	0.0000	0.0001	44	-0.0523	.9585
sIL 2Ra (pg/ml)	-0.0014	0.0010	44	-1.3117	.1964
sIL 4R (pg/ml)	0.0034	0.0014	44	2.4405	**.0188**
sIL 6R (pg/ml)	-0.0001	0.0001	44	-0.8747	.3865
sRage (pg/ml)	-0.0502	0.0216	44	-2.3234	**.0248**

sTNFRI (pg/ml)	-0.0032	0.0015	44	-2.1340	**.0385**
STNFRII (pg/ml)	-0.0008	0.0003	44	-2.4872	**.0167**
sVEGFR1 (pg/ml)	-0.0001	0.0006	44	-0.2391	.8121
sVEGFR2 (pg/ml)	-0.0003	0.0002	44	-1.7407	.0887
sVEGFR3 (pg/ml)	-0.0058	0.0022	44	-2.6162	**.0121**
sgp130 (pg/ml)	0.0000	0.0000	44	-1.7178	.0929

Ɛcc: Peak global longitudinal strain magnitude; ESV: End systolic volume; ESFS: End systolic fiber stress; BSA: Body surface area; CTnI: Cardiac Troponin I; MMP: Metalloproteinase; TIMP: Metalloproteinase inhibitor; GM.CSF: Granulocyte-macrophage colony-stimulating factor; IFNγ: Interferon γ; IL: Interlukin; sCD30: soluble CD30; sEGRF: soluble EGFR; sILR: soluble interleukin receptor; sRage: receptor for advanced glycation end products; sTNFR: soluble receptor for TNF; sVEGFR: receptors for vascular endothelial growth factor; sgp130: Glycoprotein 130

The negative sign in front of the coefficient for a specific biomarker demonstrates directionality of change in relation to a decrease in EF from V1 to V6.
